# Supplementary material for: Machine Learning-Based Peripheral Artery Disease Identification Using Laboratory-Based Gait Data
Source: Sensors (Basel). 2022 Sep 30;22(19):7432. doi: 10.3390/s22197432 (PMC9572112; doi:10.3390/s22197432)
Supplement: Supplementary file 1 [file sensors-22-07432-s001.zip › sensors-1895938-supplementary.pdf]

Machine Learning-Based Peripheral Artery Disease Identification Using Laboratory-Based Gait Signatures (Python Code)

Here, we provide a detailed step by step demonstration of data preprocessing and machine learning application to distinguish between patients with PAD and healthy controls.

Part 1 - Data Preprocessing

Python Code S1.1: Import Libraries

```
import numpy as np
import matplotlib.pyplot as plt
import pandas as pd
import seaborn as sn

from sklearn.preprocessing import LabelEncoder
from sklearn.model_selection import train_test_split
from sklearn.preprocessing import StandardScaler

from collections import Counter
from imblearn.over_sampling import SMOTE
import tensorflow as tf
import numpy
import keras
import keras.utils
from keras import utils as np_utils
from keras.models import Sequential
from keras.layers import Dense

from sklearn import svm
from sklearn import metrics
import imblearn.metrics
from keras.backend import sqrt
from math import log, pi
from sklearn.svm import SVC
from sklearn.ensemble import RandomForestClassifier
from sklearn.linear_model import LogisticRegression
from sklearn.metrics import confusion_matrix, accuracy_score

# fix random seed for reproducibility
```

```
numpy.random.seed(7)
tf.compat.v1.random.set random seed(1234)

# we use an early stop mechanisim to avoid over training model.
def earlystopping(min_delta, patience):
    es_cb = tf.keras.callbacks.EarlyStopping(monitor='loss',
                                             min_delta=min_delta,
                                             patience=patience,
                                             restore_best_weights=False)
    return es_cb
```

Python Code S1.2: Import Dataset

```
dataset = pd.read_csv('~.csv')
```

Table S1: Description of Predictive feature and data types

Data Feature Source	Feature Name	Data Feature Type
Ground Reaction Forces - Anteroposterior component	Braking peak	float
	Zero-crossing	float
	Propulsive peak	float
	Braking impulse	float
	Propulsive impulse	float
Ground Reaction Forces - Mediolateral component	Lateral peak	float
	Medial peak	float
GRF - Vertical component	Loading response peak	float
	Midstance valley	float
	Terminal stance peak	float
Ankle Joint Angle	Ankle plantarflexion maximum	float
	Ankle dorsiflexion maximum	float
Ankle Torque	Ankle dorsiflexor peak torque	float
	Ankle plantar flexor peak torque	float
Ankle Power	Early power absorption	float
	Peak power absorption	float
	Peak power generation	float
Hip Joint Angle	Hip Flexion Maximum	float
	Hip Extension Maximum	float
Hip Torque	Hip Flexor peak torque	float
	Hip Extensor peak torque	float
Hip Power	Early peak power generation	float
	Peak power absorption	float
	Peak power generation	float
Knee Joint Angle	Knee Flexion Maximum	float
	Knee Extension Maximum	float
Knee Torque	Knee Flexor peak torque	float
	Knee Extensor peak torque	float
Knee Power	Early peak power absorption	float
	Peak power generation	float
	Peak power absorption	float
Other Features	Condition	Binary

Python Code S1.3: Splitting the dataset into the Training set and Test set

```
X_train, X_test, y_train, y_test = train_test_split(X, y, test_size = 0.25, random_state =0
print("y_train: ",Counter(y_train))
print("y_test: ",Counter(y_test))
print("PAD to Healthy Ratio in the Training Set:", round(217/50, 2))
print("PAD to Healthy Ratio in the Testing Set:", round(73/16, 2))
```

```
y_train:  Counter({1: 217, 0: 50})
y_test:  Counter({1: 73, 0: 16})
PAD to Healthy Ratio in the Training Set: 4.34
PAD to Healthy Ratio in the Testing Set: 4.56
```

Here, we maintain a close ratio between healthy controls and patients with PAD in the training and testing sets. for instance, in training set the PAD to healthy ratio is 4.34, and in the testing set is 4.54.

Python Code S1.4: Feature Scaling

```
sc = StandardScaler()
X_train = sc.fit_transform(X_train)
X_test = sc.transform(X_test)
```

Python Code S1.5: SMOTE Oversampling of Training Data

To mitigate the effect of the data imbalance we apply SMOTE oversampling on the healthy controls data. This increases the sample size of healthy controls to equal the sample size of patients with PAD.

```
print("Before oversampling: ",Counter(y_train))
# define oversampling strategy
SMOTE = SMOTE()

# fit and apply the transform
X_train, y_train = SMOTE.fit_resample(X_train, y_train)

# summarize class distribution
print("After oversampling: ",Counter(y_train))
```

```
Before oversampling:  Counter({1: 217, 0: 50})
After oversampling:  Counter({0: 217, 1: 217})
```

Part 2 - Machine Learning Application

As described in the main text, we divide the data into multiple groups based on the data source (ankle, hip, knee, and GRF).

- Group 1: consists of **All** gait signatures
- Group 2: consists of **Ankle** gait signatures
- Group 3: consists of **Hip** gait signatures
- Group 4: consists of **Knee** gait signatures
- Group 5: consists of **GRF** gait signatures
- Group 6: consists of **ALL** gait signatures **except GRF**

We use four different machine learning algorithms to compare the performance:

- Neural Networks
- Support Vector Machines (SVM)
- Random Forest
- Logistic Regression

To measure the performance of each model we use several classification metrics because the data is imbalanced:

- Accuracy
- Mathew's Correlation Coefficient (MCC)
- Discriminant Power (DP)
- Geometric Mean (G-Mean)

Group 1 Model Demo

This groups consists of all predictive gait signature features, including (ankle, hip, knee, and GRF). The code can be applied to other groups, but it requires changing hyperparameters for each algorithm as listed in the Tables in each section.

Neural Networks

We fixed our neural networks architecture to have five hidden layers, the first and the last hidden layer consists of 24 neurons and the middle layers consists of 36 neurons. We set these number after applying grid search and manual tuning.

Table S2: Neural Networks Model Hyperparameters

Group Number	Category	Activation Function	Weight Initializer	Optimizer	Learning Rate
1	All features	Relu	glorot_uniform	rms	0.001
2	Ankle	tanh	glorot_uniform	adam	0.0005
3	Hip	elu	glorot_normal	rms	0.0001
4	Knee	tanh	glorot_normal	rms	0.001

5	GRF	tanh	glorot_normal	rms	0.0001
6	All except GRF	relu	glorot_normal	rms	0.001

Python Code S2.1: Neural Network Deep Learning Model Implementation

```
# Global Hyperparameters:
max_epochs = 1000
patience = 10
min_delta = 1e-5
batch_size = 10


# Group 1 Model and Hyperparameters

init_mode= 'glorot_uniform'
optimizer = tf.optimizers.RMSprop(learning_rate=0.001)
activation = 'relu'
# Model Architecture
ann = Sequential([
  Dense(units= 24 , activation=activation, kernel_initializer=init_mode), # First Hidden La
  Dense(units= 36 , activation=activation, kernel_initializer=init_mode), # Second Hidden L
  Dense(units= 36 , activation=activation, kernel_initializer=init_mode), # Third Hidden La
  Dense(units= 36 , activation=activation, kernel_initializer=init_mode), # Fourth Hideen L
  Dense(units= 24 , activation=activation, kernel_initializer=init_mode), # Fifth Hidden La

  Dense(1, activation= 'sigmoid')
])


ann.compile(loss=keras.losses.BinaryCrossentropy(from_logits=True),
            optimizer=optimizer,
            metrics=['accuracy'] )


ann_history = ann.fit(X_train, y_train,
                      epochs=max_epochs,
                      batch_size=batch_size,
                      callbacks=[earlystopping(min_delta, patience)])
```

Python Code S2.2: Neural Network Model Scores

```
y_pred = ann.predict(X_test)
y_pred = (y_pred > 0.5)

cm = confusion_matrix(y_test, y_pred)

# calculate DP
sensitivity = imblearn.metrics.sensitivity_score(y_test, y_pred)
specificity = imblearn.metrics.specificity_score(y_test, y_pred)
D = sensitivity/(1-sensitivity)
P = specificity/(1-specificity)
DP = (numpy.sqrt(3)/pi)*(log(D) + log(P))
```

```
print("Confusion Matrix:")
print(cm)
print(f"Accuracy: {metrics.accuracy_score(y_test, y_pred): .3f}")
print(f"Matthew's correlation coefficient:{metrics.matthews_corrcoef(y_test, y_pred): .3f}"
print(f"G-mean:{imblearn.metrics.geometric_mean_score(y_test, y_pred, average='binary'): .3
print(f"DP:{DP: .3f}")
```

Output:

```
Confusion Matrix:
[[12  4]
 [ 6 67]]
Accuracy:  0.888
Matthew's correlation coefficient: 0.638
G-mean: 0.830
DP: 1.936
```

SVM

Table S3: SVM Model Hyperparameters				
Group Number	Category	Regularization parameter (C)	Kernel	Kernel coefficient
1	All features	0.8	linear	
2	Ankle	0.75	rbf	0.8
3	Hip	1	rbf	0.9
4	Knee	1	rbf	0.9
5	GRF	1	rbf	0.8
6	All except GRF	1	rbf	0.3

Python Code S2.3: SVM Machine Learning Model Implementation

```
SVM_model = SVC(kernel = 'linear' , C=1 ,probability=True)
SVM_model.fit(X_train, y_train)
y_pred_svm = SVM_model.predict(X_test)
```

Python Code S2.4: SVM Model Scores

```
cm = confusion_matrix(y_test, y_pred_svm)
# calculate DP
sensitivity = imblearn.metrics.sensitivity_score(y_test, y_pred_svm)
specificity = imblearn.metrics.specificity_score(y_test, y_pred_svm)
D = sensitivity/(1-sensitivity)
P = specificity/(1-specificity)
DP = (numpy.sqrt(3)/pi)*(log(D) + log(P))

print("Confusion Matrix:")
print(cm)
print(f"Accuracy: {metrics.accuracy_score(y_test, y_pred_svm): .3f}")
print(f"Matthew's correlation coefficient:{metrics.matthews_corrcoef(y_test, y_pred_svm): .
print(f"G-mean:{imblearn.metrics.geometric_mean_score(y_test, y_pred_svm, average='binary')
print(f"DP:{DP: .3f}")
```

Output:

```
Confusion Matrix:
[[12  4]
 [21 52]]
Accuracy:  0.719
Matthew's correlation coefficient: 0.368
G-mean: 0.731
DP: 1.106\
```

Random Forrest

Table S4: Random Forest Model Hyperparameters				
Group Number	Category	number of trees	criterion	maximum depth of the tree
1	All features	250	gini	20
2	Ankle	125	gini	50
3	Hip	250	gini	50
4	Knee	25	entropy	20
5	GRF	125	gini	50
6	All except GRF	125	gini	50

Python Code S2.5: Random Forest Machine Learning Model Implementation

```
RF_model = RandomForestClassifier(n_estimators = 250, criterion = 'gini', max_depth=20)
RF_model.fit(X_train, y_train)
y_pred_rf = RF_model.predict(X_test)
```

Python Code S2.6: Random Forest Model Scores

```
cm = confusion_matrix(y_test, y_pred_rf)
# calculate DP
sensitivity = imblearn.metrics.sensitivity_score(y_test, y_pred_rf)
specificity = imblearn.metrics.specificity_score(y_test, y_pred_rf)
D = sensitivity/(1-sensitivity)
P = specificity/(1-specificity)
DP = (numpy.sqrt(3)/pi)*(log(D) + log(P))

print("Confusion Matrix:")
print(cm)
print(f"Accuracy: {metrics.accuracy_score(y_test, y_pred_rf): .3f}")
print(f"Matthew's correlation coefficient:{metrics.matthews_corrcoef(y_test, y_pred_rf): .3
print(f"G-mean:{imblearn.metrics.geometric_mean_score(y_test, y_pred_rf, average='binary'):
print(f"DP:{DP: .3f}")
```

Output:

```
Confusion Matrix:
[[12  4]
 [ 6 67]]
Accuracy:  0.888
Matthew's correlation coefficient: 0.638
G-mean: 0.830
DP: 1.936
```

Logistic Regression

Python Code S2.7: Logistic Regression Machine Learning Model Implementation

```
classifier = LogisticRegression(random_state = 0)
classifier.fit(X_train, y_train)
y_pred_lg = classifier.predict(X_test)
```

Python Code S2.8: Logistic Regression Model Scores

```
cm = confusion_matrix(y_test, y_pred_lg)
# calculate DP
sensitivity = imblearn.metrics.sensitivity_score(y_test, y_pred_lg)
specificity = imblearn.metrics.specificity_score(y_test, y_pred_lg)
D = sensitivity/(1-sensitivity)
P = specificity/(1-specificity)
DP = (numpy.sqrt(3)/pi)*(log(D) + log(P))

print("Confusion Matrix:")
print(cm)
print(f"Accuracy: {metrics.accuracy_score(y_test, y_pred_lg): .3f}")
print(f"Matthew's correlation coefficient:{metrics.matthews_corrcoef(y_test, y_pred_lg): .3
print(f"G-mean:{imblearn.metrics.geometric_mean_score(y_test, y_pred_lg, average='binary'):
print(f"AGF:{AGF: .3f}")
print(f"DP:{DP: .3f}")
```

Output:

```
Confusion Matrix:
[[14  2]
 [19 54]]
Accuracy:  0.764
Matthew's correlation coefficient: 0.489
G-mean: 0.805
AGF: 0.931
DP: 1.649
```

Group1 Results Summary

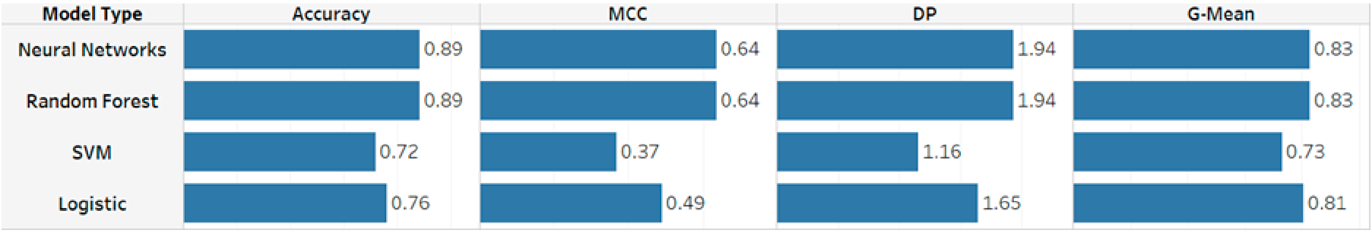

Figure S1: Summary of Models Results

Summary of All Machine Learning models Results

Table S5: Performance Summary of All Machine Learning Models							
Metric	Model Type	Group Category					
Category		All	Ankle	Hip	Knee	GRF	Ankle, Hip, Knee
Group Number		Group 1	Group 2	Group 3	Group 4	Group 5	Group 6
Accuracy	Neural Networks	0.89	0.79	0.78	0.81	0.82	0.84
	Random Forest	0.89	0.69	0.73	0.75	0.87	0.83
	SVM	0.72	0.76	0.80	0.75	0.73	0.82
DP	Neural Networks	1.94	0.95	0.82	0.90	1.87	1.33
	Random Forest	1.94	0.64	0.29	0.71	2.09	1.19
	SVM	1.16	-0.16	0.63	0.08	1.01	0.74
G-Mean	Neural Networks	0.83	0.65	0.61	0.54	0.84	0.84
	Random Forest	0.83	0.63	0.46	0.60	0.87	0.63
	SVM	0.73	0.24	0.42	0.33	0.71	<< 0
MCC	Neural Networks	0.64	0.33	0.27	0.27	0.57	0.44
	Random Forest	0.64	0.22	0.09	0.24	0.64	0.39
	SVM	0.37	-0.03	0.16	0.02	0.35	0.00
Best Model		Neural Networks, Random Forest	Neural Networks	Neural Networks	Neural Networks	Random Forest	Neural Networks
